# ISSR diversity in *Jatropha curcas* germplasm and offspring of selected parentals

**DOI:** 10.1016/j.dib.2018.08.102

**Published:** 2018-08-30

**Authors:** Juan Ubaldo Sánchez-Velázquez, Ana Ramos-Díaz

**Affiliations:** Centro de Investigación y Asistencia en Tecnología y Diseño del Estado de Jalisco A. C., Sede Sureste, Tablaje Catastral 31264 Km 5.5 Carretera Sierra Papacal-Chuburna Puerto, Parque Científico Tecnológico de Yucatán, CP: 97302 Mérida, Yucatán, Mexico

## Abstract

This article contains metadata related to the research article “Behavior of genetic diversity in F1 crosses of selected accessions of *Jatropha curcas*” (Sánchez-Velázquez et al., 2018). The data presented in this article belong to a diversity study using ISSR molecular markers of a *J. curcas* germplasm collection that includes bred offspring and analysis of similarity between accessions. We tested previously reported primers in PCR assays to obtain a genetic profile of the accessions. These profiles were used to calculate Dice similarities. Similarity between offspring and parentals can be compared either with the maternal side or paternal side.

**Specifications Table**

**Table:** 

Subject area	*Agronomy*
More specific subject area	*Molecular biology to assist plant breeding*
Type of data	*A table with primer information, a binary matrix in a table, and a similarity index matrix in a table*
How data was acquired	*Amplification of molecular markers through PCR and software assistance.*
Data format	*Filtered*
Experimental factors	*We test 25 primers previously reported using PCR assays, and selected those with better reproducibility. PCR products of each primer were separated on agarose gel, photo documented with UV light and digitized. Digital images with banding profiles were analyzed with the software GelAnalyzer 2010a and banding profiles were transformed to a binary matrix containing the presence/absence of each allele amplified by selected ISSR primers.*
Experimental features	*Among a Jatropha germplasm, six parentals were selected for a breeding program, and three accessions were selected for control purpose. Parentals were tested as maternal receptor or paternal donor, and 15 crosses were obtained. All accessions were evaluated with ISSR primers and Dice similarities of each accession were compared with each other and clustered using the UPGMA methodology.*
Data source location	*An experimental plantation was established at CIATEJ-Sureste, Km 5.5 on Road Sierra Papacal – Port Chuburna, Sierra Papacal, Mérida, Yucatán, México*
Data accessibility	*With this article*
Related research article	*J. U. Sánchez-Velázquez, Pacheco-López N. A., López-Puc G., Ramos-Díaz A. Behavior of genetic diversity in F1 crosses of selected accessions of J. curcas. Industrial Crops and Products (in press).*

**Value of the Data**

● Data presented here show different polymorphic alleles and frequencies in accessions of *J. curcas* than previously reported for the same primers by Gupta et al. [2].
● Similarity between crosses and parental can be appreciated to perform better decisions in breeding programs.
● This is the first report of where similarity of a parental can be compared with its offspring when used as maternal receptor or a paternal donor in a *J. curcas* breeding program.
● These data can be compared with future advances in the breeding program of *J. curcas* and can be used to select the best parentals for F2 and further crosses.

## 1 Data

We present three tables of data related to the research article of Sánchez-Velázquez et al. [1]. The first table presents the primers used and the number of bands amplified by each primer. The second table is the presence/absence matrix of alleles amplified by the ISSR markers. These data were obtained after the testing of 25 primers and selecting only those with reproducibility in at least three independent repetitions of the assay. The third table shows the Dice similarity index among accessions; these data were obtained by computing the Dice index between all possible pairs of accessions including parental vs parental, parental vs control accessions, parental vs crosses, control accessions vs crosses and crosses vs crosses with the software PAST 3.17 [3]. Note that a new version of the software is available, PAST 3.2, that uses the same algorithm to compute the Dice index for binary data (Table 1, Table 2, Table 3).

**Table 1:** Primers used and number of alleles amplified by each primer.

**Primer**	**No. of total bands**	**No. of polymorphic bands**	**No. of reported**^a^**polymorphic bands**
ISSR2^a^	13	4	3
ISSR3^a^	10	4	7
ISSR7^a^	4	2	2
ISSR10^a^	12	10	3
ISSR24^a^	4	2	6
ISSR25^a^	8	5	7
Total	51	27	28

a [2].

**Table 2:** Binary matrix of presence/absence of alleles amplified by selected primers.

Accessions	ISSR2													ISSR3											ISSR7				ISSR10												ISSR24				ISSR 25							
Alleles	1	2	3	4	5	6	7	8	9	10	11	12	13	1	2	3	4	5	6	7	8	9	10	11	1	2	3	4	1	2	3	4	5	6	7	8	9	10	11	12	1	2	3	4	1	2	3	4	5	6	7	8
JatroP1	0	0	1	1	1	0	0	1	1	1	1	1	1	1	0	1	1	1	0	1	1	1	1	1	1	1	1	1	0	0	0	0	0	1	0	0	0	0	1	0	0	1	1	0	1	1	1	1	1	1	1	1
JatroP2	0	0	1	1	1	1	0	1	1	1	1	1	1	1	0	1	1	1	1	1	1	1	1	1	1	1	1	1	0	0	1	0	1	1	1	0	0	1	1	1	0	1	1	0	1	0	1	1	1	1	1	1
JatroP3	0	1	1	1	1	1	1	1	1	1	1	1	1	1	0	1	1	1	0	1	1	1	1	1	1	1	1	1	1	0	1	0	1	1	0	1	1	1	1	1	0	1	1	0	1	0	1	1	1	1	1	1
JatroP4	0	1	1	1	1	0	1	1	1	1	1	1	1	1	0	1	1	1	0	1	1	1	1	1	1	1	1	1	1	0	1	1	1	1	1	1	0	1	1	1	0	1	1	1	1	0	1	0	1	1	1	0
JatroP5	0	0	1	1	1	0	1	1	1	1	1	1	1	1	1	1	1	1	0	0	1	1	1	1	1	1	1	1	1	1	1	1	1	1	1	1	1	1	1	1	1	1	1	1	1	1	1	1	1	0	1	1
JatroP6	0	0	1	1	1	0	1	1	1	1	1	1	1	1	1	1	1	1	0	0	1	1	1	1	1	1	1	1	0	1	1	0	1	1	1	1	1	1	1	1	0	1	1	1	1	1	1	1	1	1	1	1
JatroT1	0	0	1	1	1	0	1	1	1	1	1	1	1	1	0	1	1	1	1	1	1	1	1	1	1	1	1	1	1	1	1	1	1	1	1	1	0	1	1	1	1	1	1	0	1	1	1	1	1	1	1	1
JatroT2	0	1	1	1	1	0	1	1	1	1	1	1	1	1	1	1	1	1	1	1	1	1	1	1	1	1	1	1	1	1	1	1	1	1	1	1	1	1	1	1	1	1	1	0	1	1	1	1	0	1	1	1
JatroT3	1	0	1	1	1	1	1	1	1	1	1	1	1	1	1	1	1	1	1	1	1	1	1	1	1	1	1	1	1	1	1	0	1	1	1	1	0	1	1	1	1	1	1	1	1	0	1	0	0	0	1	0
JatroC1-1Fx2P	1	0	1	1	1	1	1	1	1	1	1	1	1	1	0	1	1	1	1	1	1	1	1	1	1	1	1	1	0	0	1	1	0	1	0	0	1	1	1	1	0	1	1	0	1	0	1	1	1	1	1	0
JatroC2–5Fx2P	0	0	1	1	1	0	0	1	1	1	1	1	1	1	0	1	1	1	0	0	1	1	1	1	1	1	0	1	0	1	1	0	1	1	1	1	1	1	1	1	1	1	1	1	1	1	1	1	1	1	1	1
JatroC3-2Fx4P	0	0	1	1	1	1	1	1	1	1	1	1	1	1	0	1	1	1	1	1	1	1	1	1	1	1	1	1	0	0	0	1	0	1	1	0	1	1	1	1	1	1	1	1	1	1	1	0	0	1	1	1
JatroC4-1Fx4P	1	0	1	1	1	1	1	1	1	1	1	1	1	1	1	1	1	1	1	1	1	1	1	1	1	1	1	1	1	1	1	1	1	1	1	1	1	1	1	1	1	1	1	1	1	1	1	0	1	1	1	1
JatroC5-1Fx5P	1	0	1	1	1	1	1	1	1	1	1	1	1	1	1	1	1	1	1	0	1	1	1	1	1	1	1	1	1	1	1	1	1	1	1	1	1	1	1	1	0	1	1	1	1	0	1	1	0	0	1	1
JatroC6-2Fx6P	1	0	1	1	1	0	0	1	1	1	1	1	1	1	1	1	1	1	1	1	1	1	1	1	1	1	1	1	1	1	1	1	1	1	1	1	1	1	1	1	1	1	1	1	1	0	1	0	1	1	1	1
JatroC7-1Fx6P	1	0	1	1	1	0	0	1	1	1	1	1	1	0	1	1	1	1	1	0	1	1	1	1	1	1	1	1	0	1	1	1	1	1	1	1	1	1	1	1	1	1	1	1	1	0	1	1	1	1	1	1
JatroC8-2Fx3P	1	0	1	1	1	1	0	1	1	1	1	1	1	0	1	1	1	1	0	1	1	1	1	1	1	1	0	0	1	1	1	1	1	1	1	1	1	1	1	1	1	1	1	1	1	0	1	1	0	1	1	1
JatroC9-1Fx3P	1	0	1	1	1	1	1	1	1	1	1	1	1	1	1	1	1	1	0	1	1	1	1	1	1	1	1	1	0	1	1	1	0	1	1	1	1	1	1	1	0	1	1	0	1	1	1	0	1	1	1	1
JatroC10-4Fx5P	1	1	1	1	1	0	1	1	1	1	1	1	1	1	1	1	1	1	0	1	1	1	1	1	1	1	1	1	1	1	1	1	1	1	1	1	0	1	1	1	1	1	1	1	1	0	1	0	0	0	1	0
JatroC11-4Fx6P	1	1	1	1	1	1	1	1	1	1	1	1	1	1	1	1	1	1	0	1	1	1	1	1	1	1	1	1	0	1	1	1	1	1	1	1	1	1	1	1	1	1	1	0	1	0	1	0	1	1	1	1
JatroC12-4Fx3P	1	1	1	1	1	1	0	1	1	1	1	1	1	1	0	1	1	1	1	0	1	1	1	1	1	1	1	1	1	1	1	1	1	1	1	0	0	1	1	1	0	1	1	1	1	0	1	0	0	0	1	0
JatroC13-5Fx6P	0	1	1	1	1	0	0	1	1	1	1	1	1	1	0	1	1	1	0	1	1	1	1	1	1	1	1	0	1	1	1	1	1	1	1	1	1	1	1	1	1	1	1	0	1	0	1	0	0	0	1	0
JatroC14-5Fx3P	1	1	1	1	1	1	1	1	1	1	1	1	1	1	1	1	1	1	0	1	1	1	1	1	1	1	1	1	0	1	1	0	1	1	1	1	1	1	1	1	0	1	1	1	1	0	1	0	0	0	1	1
JatroC15-6Fx3P	0	0	1	1	1	0	0	1	1	1	1	1	1	1	1	1	1	1	0	0	1	1	1	1	1	1	1	1	0	1	1	0	0	1	0	0	1	1	1	1	0	1	1	1	1	0	1	0	0	0	1	0

**Table 3:** Similarity values between pairs of accessions.

	JatroP1	JatroP2	JatroP3	JatroP4	JatroP5	JatroP6	JatroT1	JatroT2	JatroT3	JatroC1-1Fx2P	JatroC2–5Fx2P	JatroC3-2Fx4P
JatroP1	1	0.88888889	0.85333333	0.81081081	0.79487179	0.84210526	0.84615385	0.8	0.73684211	0.86111111	0.83783784	0.83783784
JatroP2	0.88888889	1	0.91358025	0.875	0.83333333	0.87804878	0.9047619	0.86046512	0.85365854	0.8974359	0.875	0.875
JatroP3	0.85333333	0.91358025	1	0.91566265	0.87356322	0.89411765	0.89655172	0.8988764	0.84705882	0.91358025	0.86746988	0.84337349
JatroP4	0.81081081	0.875	0.91566265	1	0.88372093	0.88095238	0.90697674	0.88636364	0.88095238	0.875	0.85365854	0.85365854
JatroP5	0.79487179	0.83333333	0.87356322	0.88372093	1	0.95454545	0.93333333	0.93478261	0.88636364	0.83333333	0.93023256	0.86046512
JatroP6	0.84210526	0.87804878	0.89411765	0.88095238	0.95454545	1	0.90909091	0.91111111	0.86046512	0.85365854	0.95238095	0.85714286
JatroT1	0.84615385	0.9047619	0.89655172	0.90697674	0.93333333	0.90909091	1	0.95652174	0.88636364	0.85714286	0.90697674	0.88372093
JatroT2	0.8	0.86046512	0.8988764	0.88636364	0.93478261	0.91111111	0.95652174	1	0.88888889	0.8372093	0.88636364	0.88636364
JatroT3	0.73684211	0.85365854	0.84705882	0.88095238	0.88636364	0.86046512	0.88636364	0.88888889	1	0.82926829	0.83333333	0.85714286
JatroC1-1Fx2P	0.86111111	0.8974359	0.91358025	0.875	0.83333333	0.85365854	0.85714286	0.8372093	0.82926829	1	0.825	0.875
JatroC2–5Fx2P	0.83783784	0.875	0.86746988	0.85365854	0.93023256	0.95238095	0.90697674	0.88636364	0.83333333	0.825	1	0.85365854
JatroC3-2Fx4P	0.83783784	0.875	0.84337349	0.85365854	0.86046512	0.85714286	0.88372093	0.88636364	0.85714286	0.875	0.85365854	1
JatroC4-1Fx4P	0.7804878	0.86363636	0.87912088	0.88888889	0.93617021	0.91304348	0.93617021	0.9375	0.93478261	0.86363636	0.88888889	0.91111111
JatroC5-1Fx5P	0.74358974	0.85714286	0.87356322	0.86046512	0.93333333	0.90909091	0.88888889	0.91304348	0.93181818	0.85714286	0.86046512	0.86046512
JatroC6-2Fx6P	0.78481013	0.87058824	0.86363636	0.89655172	0.92307692	0.8988764	0.92307692	0.92473118	0.92134831	0.84705882	0.89655172	0.87356322
JatroC7-1Fx6P	0.80519481	0.86746988	0.86046512	0.87058824	0.94382022	0.94252874	0.8988764	0.9010989	0.87356322	0.86746988	0.94117647	0.84705882
JatroC8-2Fx3P	0.72727273	0.84337349	0.8372093	0.82352941	0.87640449	0.85057471	0.87640449	0.9010989	0.89655172	0.81927711	0.87058824	0.84705882
JatroC9-1Fx3P	0.80519481	0.86746988	0.86046512	0.84705882	0.87640449	0.89655172	0.8988764	0.9010989	0.87356322	0.89156627	0.84705882	0.89411765
JatroC10-4Fx5P	0.73684211	0.80487805	0.84705882	0.92857143	0.90909091	0.86046512	0.88636364	0.91111111	0.95348837	0.82926829	0.83333333	0.83333333
JatroC11-4Fx6P	0.78481013	0.87058824	0.90909091	0.89655172	0.9010989	0.8988764	0.9010989	0.92473118	0.8988764	0.89411765	0.87356322	0.87356322
JatroC12-4Fx3P	0.73972603	0.86075949	0.82926829	0.88888889	0.84705882	0.81927711	0.84705882	0.85057471	0.91566265	0.83544304	0.81481481	0.83950617
JatroC13-5Fx6P	0.75	0.82051282	0.86419753	0.9	0.88095238	0.82926829	0.88095238	0.90697674	0.87804878	0.82051282	0.85	0.825
JatroC14-5Fx3P	0.76315789	0.85365854	0.89411765	0.88095238	0.88636364	0.90697674	0.84090909	0.88888889	0.93023256	0.85365854	0.85714286	0.85714286
JatroC15-6Fx3P	0.80597015	0.82191781	0.81578947	0.82666667	0.86075949	0.88311688	0.78481013	0.81481481	0.85714286	0.84931507	0.85333333	0.82666667

## 2 Experimental design, materials, and methods

### 2.1 Plant material

An experimental plantation of 24 accessions of *J. curcas* was established at CIATEJ. The plant material was donate by “Agroindustria Alternativa del Sureste SPR de RL de CV”. The germplasm collection is composed of 6 parental accessions, 3 control accession and 15 selected crosses. Parentals can serve as maternal receptor or paternal donor. All accessions were propagated vegetatively by stakes. Leaf samples (0.5 g) were taken and frozen in liquid nitrogen and maintained at −80 °C for molecular marker analysis at the moment of DNA extraction.

### 2.2 DNA extraction

Genomic DNA extraction was performed following the CTAB protocol [4], with modifications. The lysis solution consisted of 100 mM of Tris (pH 8), 20 mM of EDTA, 1.6 M of NaCl, 2% CTAB, 2.5% PVPP and 0.5% ß-mercaptoethanol. The solution was preheated to 60 °C for 30 min, and then the leaf material was macerated in the lysis solution (0.1 g of leaf per ml of lysis solution). The macerated material was transferred to a new microcentrifuge tube and incubated for 30 min at 60 °C with continuous inversions, then allowed to cool to room temperature. Afterwards, 500 µl of chloroform-alcohol isoamyl were added and mixed by inversion until the mixture was emulsified. It was then centrifuged at 8000*g* for 15 min and the upper phase was separated and transferred into a new tube. One hundred and thirty microliters of precipitate solution from the GeneElute Plant Genomic DNA mini prep kit (SIGMA-ALDRICH®) were then added to the mixture, which was transferred to the kit׳s filtration column and centrifuged; afterwards the column was discarded. After adding 700 µl of the kit׳s bonding solution, the mixture was transferred to the DNA retention column, where it was centrifuged, and the filtrate was discarded. Finally, two washes were performed with the kit׳s washing solution and the DNA was eluted in 100 µl of deionized water. The extracted DNA was quantified by spectroscopy and its quality was checked by measuring the A260/A280 absorbance, looking for an absorbance ratio between 1.8 and 2.0.

### 2.3 Amplification and characterization of molecular markers

A PCR procedure was implemented to 25 primers [2] in triplicate with the gDNA template from leaf samples, using the following program: 5 min at 95 °C for the DNA denaturation step, 40 cycles of 1 min at 95 °C, 1 min at 45 °C and 2 min at 72 °C for the ISSR fragment amplification step, with a final elongation step for 10 min at 72 °C.

The PCR products were separated by electrophoresis in a 1% agarose gel under 0.4 V/cm^2^ of gel area and stained with ethidium bromide at a concentration of 0.05 µg/ml and visualized under UV light. The banding pattern of accessions were digitized and characterized. Only banding from primers with at least triplicate repeatability were considered for further analysis.

For banding pattern characterization, digitized banding profiles were analyzed using the software GelAnalyzer 2010a. The molecular marker 1Kb plus DNA ladder (Invitrogen™) was used to compare the molecular weight of alleles amplified by each primer on all accessions, and a position for every allele was assigned. Then the specific allele profile of each accession was converted to a presence/absence matrix of all possible positions.

### 2.4 Similarity calculation

Similarity was calculated by computing the Dice index between all pairs of possible combinations of accessions with the software PAST 3.17 [3], according to:

$$D_{\mathrm{jk}}=2M/\left(2M+N\right)$$

where *M* is the total number of allele matches between a pair of accessions and *N* the total number of possible alleles between all accessions.
