# Supplementary material for: ISSR diversity in Jatropha curcas germplasm and offspring of selected parentals
Source: Data Brief. 2018 Aug 30;20:761–6. doi: 10.1016/j.dib.2018.08.102 (PMC6129730; doi:10.1016/j.dib.2018.08.102)
Supplement: Supplementary file 2 — Supplementary material [file mmc2.docx]

**Conflict of interest**

Authors declare no conflict of interest
